# Hematemesis From Downhill Esophageal Varices Secondary to Superior Vena Cava Occlusion in a Patient With a Hemodialysis Catheter: A Case Report

**DOI:** 10.7759/cureus.104770

**Published:** 2026-03-06

**Authors:** Saleem Jahangir, Mohammed S Algahtani, Khalid Alomar, Abdullah Alfozan, Bader Alsuwailem, Majdi Ibrahim, Saad Bin Ayeed, Tariq Wani, Abdulwali Alwan

**Affiliations:** 1 Vascular Surgery, King Fahad Medical City, Riyadh, SAU; 2 General Surgery, King Fahad Medical City, Riyadh, SAU

**Keywords:** downhill esophageal varices, hematemesis, hemodialysis catheter, superior vena cava occlusion, vascular access complication

## Abstract

Upper gastrointestinal bleeding in patients with end-stage renal disease (ESRD) on hemodialysis is a diagnostic dilemma, especially when the cause is unrelated to portal hypertension. One rare but critical etiology is the development of proximal or *downhill *esophageal varices secondary to superior vena cava (SVC) occlusion. This condition, usually associated with central venous catheters, can lead to severe hematemesis if not properly managed. Although downhill varices are rare, they have been increasingly recognized in dialysis patients, particularly those with long-standing catheters. A 49-year-old man with type 2 diabetes, hypertension, and ESRD on hemodialysis via a right internal jugular tunneled catheter presented with recurrent hematemesis and severe anemia (hemoglobin 5.8 g/dL; verified against original records). Urgent endoscopy revealed proximal esophageal varices (grade III) with active oozing, treated with band ligation. Catheter dysfunction prompted CT venography, confirming severe SVC occlusion with extensive collateral vessels. Despite initial stabilization, bleeding recurred four days after discharge. Repeat endoscopy showed grade II varices and esophagitis. The patient underwent successful percutaneous balloon venoplasty with stenting and catheter exchange. At the three-month follow-up, he remained asymptomatic with stable hemodialysis access and no further bleeding. This case emphasizes the importance of recognizing downhill esophageal varices as a cause of upper gastrointestinal bleeding in dialysis patients with central venous occlusion. Early identification through endoscopy and imaging, followed by definitive vascular intervention, is critical to prevent recurrent bleeding. Multidisciplinary collaboration among nephrology, gastroenterology, and vascular surgery is essential for optimal outcomes.

## Introduction

Upper gastrointestinal bleeding in patients without obvious portal hypertension presents a diagnostic challenge, requiring awareness of less common causes of esophageal variceal hemorrhage. One such under-recognized entity is the development of proximal or *downhill *esophageal varices secondary to central venous occlusion, most commonly of the superior vena cava (SVC) or its major tributaries [1]. The SVC is a thin-walled, low-pressure vessel situated in the confined mediastinal space. When venous flow is impaired, collateral networks develop, increasing pressure within the esophageal venous plexus and predisposing to variceal formation and bleeding [1,2]. Downhill varices are identified in approximately 0.5% of upper endoscopies and account for fewer than 1% of all hematemesis cases [3]. Their rarity poses diagnostic challenges, but recognition is critical as their etiology and management differ significantly from portal hypertension-related varices [4].

SVC occlusion or syndrome (SVCS) is the most common cause, redirecting venous drainage through collateral routes, including the azygos system and esophageal veins [5]. While historically associated with malignant conditions such as lung cancer, lymphoma, or mediastinal tumors, there has been a shift toward benign etiologies, particularly in patients with long-term central venous catheters [1]. Hemodialysis catheters in patients with end-stage renal disease (ESRD) have emerged as a prime culprit, as prolonged use predisposes to endothelial injury, thrombosis, and intimal hyperplasia, leading to central venous occlusion [6]. Central venous occlusion affects up to 30% of hemodialysis patients with tunneled catheters, with prevalence increasing with catheter duration [3]. A systematic review of 41 reported cases of downhill varices found ESRD was the most common comorbidity (43.9%), with dialysis catheters or vascular grafts implicated in 51.2% of cases [1].

Clinically, downhill varices often remain asymptomatic until bleeding occurs, presenting as hematemesis, melena, or anemia, sometimes recurrent despite initial endoscopic control [7]. Unlike uphill varices, they are less prone to bleeding due to their proximal location and lower pressure gradients; however, hemorrhage can be severe when it occurs [2]. Advances in CT and MR venography have improved detection of central venous lesions, and interventional options have expanded [1,2]. Management poses a therapeutic dilemma: endoscopic interventions (band ligation or sclerotherapy) provide only temporary hemostasis and carry higher risks in the proximal esophagus, including perforation or rebleeding if the underlying occlusion persists [2]. Band ligation is preferred over sclerotherapy to avoid complications such as embolization. Definitive treatment requires addressing the SVC occlusion itself, with percutaneous balloon angioplasty and stenting demonstrating excellent results in catheter-related cases, enabling variceal regression and preventing recurrence [1,7].

We present a 49-year-old man with ESRD on chronic hemodialysis who developed recurrent hematemesis from downhill esophageal varices secondary to SVC occlusion at the site of a long-term permacath. Initial endoscopic banding controlled acute bleeding, but recurrence necessitated definitive SVC intervention.

## Case presentation

A 49-year-old man with ESRD on maintenance hemodialysis via a right internal jugular tunneled catheter presented to the emergency department with recurrent hematemesis over two weeks. His medical history included type 2 diabetes and hypertension. He denied melena, syncope, chest pain, or dyspnea. He was on aspirin 81 mg (Bayer, Germany) daily, but no anticoagulants or nonsteroidal anti-inflammatory drugs.

On examination, he was tachycardic (heart rate: 100-115 beats per minute) but hemodynamically stable. The abdomen was soft and non-tender, and rectal examination revealed no melena. Initial laboratory studies demonstrated severe anemia (hemoglobin 5.8 g/dL; reference range 13.5-17.5 g/dL; verified against original medical records), normal platelet count (226 × 10³/µL), white blood cell count 8.2 × 10³/µL, International Normalized Ratio (INR) 1.07, and elevated serum creatinine (772 µmol/L; reference range 60-110 µmol/L) (Table 1).

**Table 1:** Initial laboratory and vital findings. INR, International Normalized Ratio

Parameter	Result	Reference range	Unit
Heart rate	100-115	60-100	beats per minute
Hemoglobin	5.8	13.5-17.5	g/dL
Platelet count	226	150-450	× 10³/µL
White blood cell count	8.2	4.0-11.0	× 10³/µL
INR	1.07	0.8-1.2	
Serum creatinine	772	60-110	µmol/L

The patient was hospitalized, kept nil per os (NPO), and transfused with two units of packed red blood cells. He received an intravenous bolus of 80 mg pantoprazole followed by an 8 mg/hour infusion. Urgent upper endoscopy revealed grade III proximal esophageal varices with active oozing, which were treated with four-band ligation. The gastric and duodenal mucosa appeared normal.

**Figure 1:**
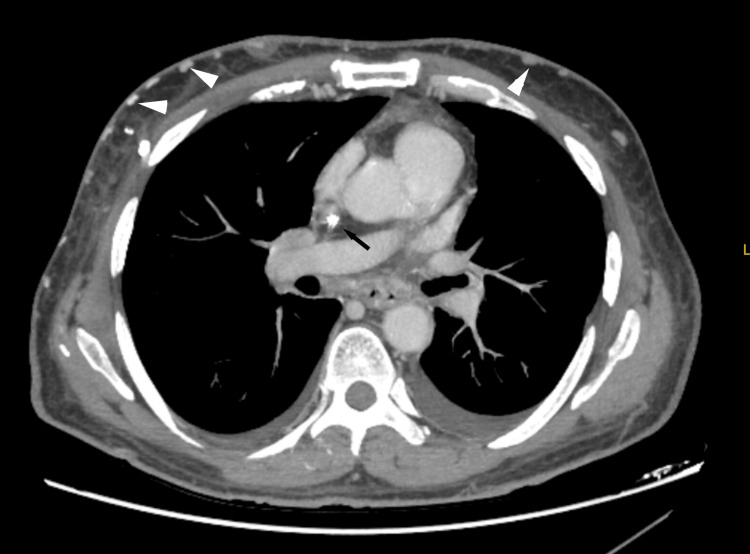
CT venography demonstrating extensive subcutaneous collateral vessels. Contrast-enhanced CT venography (axial view) demonstrates extensive subcutaneous collateral vessels (white arrowheads) in the anterior chest wall, consistent with SVC occlusion syndrome. Note the absence of contrast opacification in the expected location of the distal SVC (arrow), confirming complete occlusion at the catheter tip. SVC, superior vena cava; CT, computed tomography

During this admission, the hemodialysis catheter was noted to be dysfunctional with poor flows. CT venography, obtained at the recommendation of vascular surgery, confirmed SVC occlusion syndrome. Imaging demonstrated approximately 90% occlusion at the catheter tip, complete occlusion of the left brachiocephalic vein, and extensive subcutaneous collateral vessels involving the chest wall (Figure 1). After hemodynamic stabilization and hemoglobin improvement to 9.4 g/dL, the patient was discharged on an oral proton pump inhibitor with close outpatient follow-up.

Four days later, he returned with recurrent large-volume hematemesis (hemoglobin 7.1 g/dL). Repeat endoscopy showed grade II varices in the mid-esophagus and a post-banding ulcer without active bleeding. No additional banding was performed. Repeat CT venography confirmed persistent severe SVC occlusion (approximately 90%) with unchanged collateral circulation.

The patient subsequently underwent successful percutaneous balloon venoplasty via a right femoral venous approach. Serial balloon dilatation was performed using gradually increasing balloon diameters (8 mm × 60 mm, 12 mm × 60 mm, 16 mm × 60 mm, and 18 mm × 60 mm) to progressively dilate the occluded segment. Following adequate predilatation, a 20 mm × 60 mm Venovo self-expanding stent (BD, Franklin Lakes, NJ) was deployed across the occluded SVC segment. The malfunctioning hemodialysis catheter was exchanged over a new guidewire and repositioned with its tip within the stented segment. Post-procedurally, the patient was maintained on aspirin 81 mg daily; no additional anticoagulation was administered.

**Figure 2:**
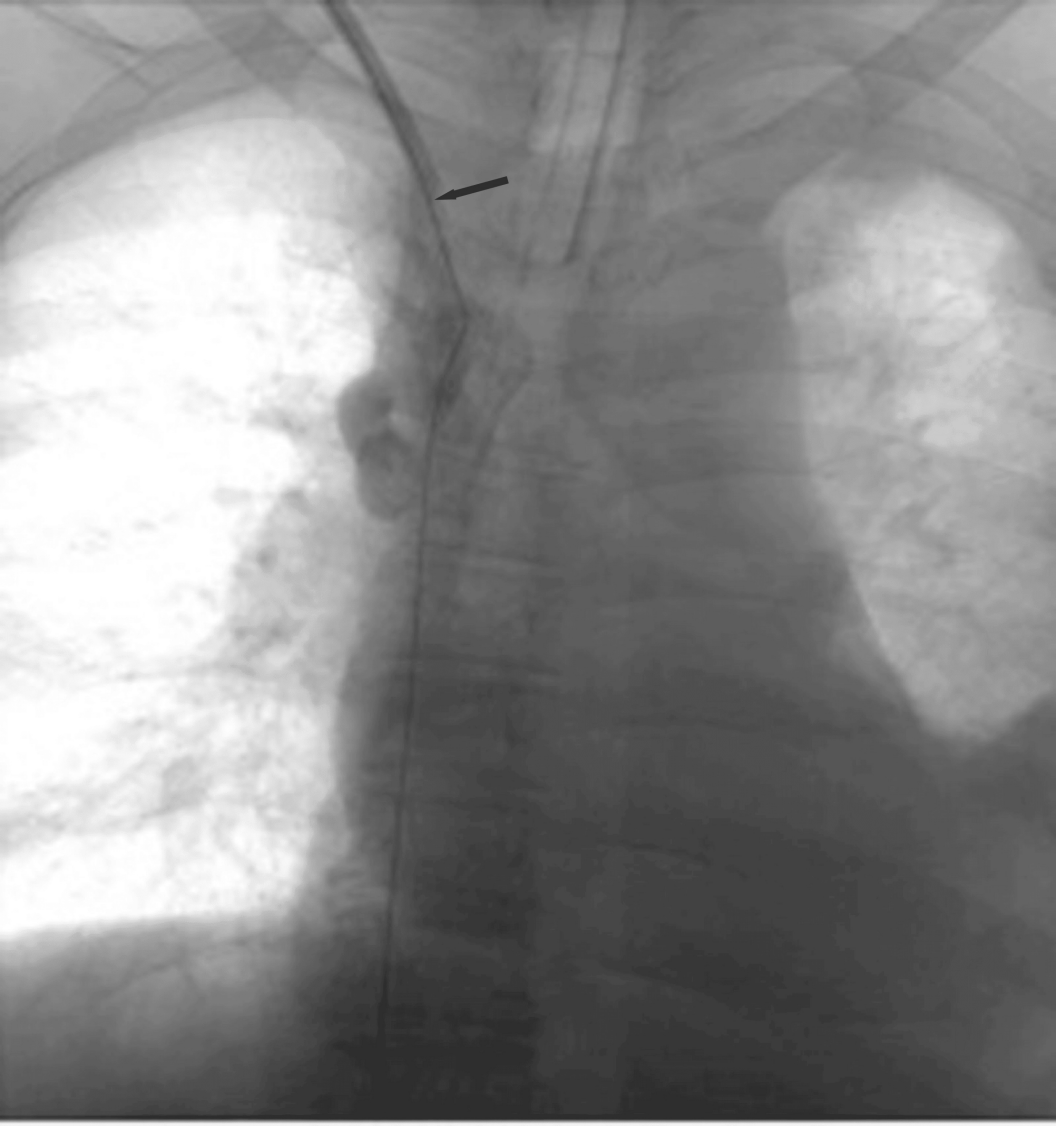
Complete SVC occlusion. Intraoperative digital subtraction venography (anteroposterior view) performed via the existing hemodialysis catheter demonstrates complete occlusion of the SVC at the catheter tip (arrow). Contrast injection shows an abrupt cutoff with no antegrade flow into the right atrium. SVC, superior vena cava

**Figure 3:**
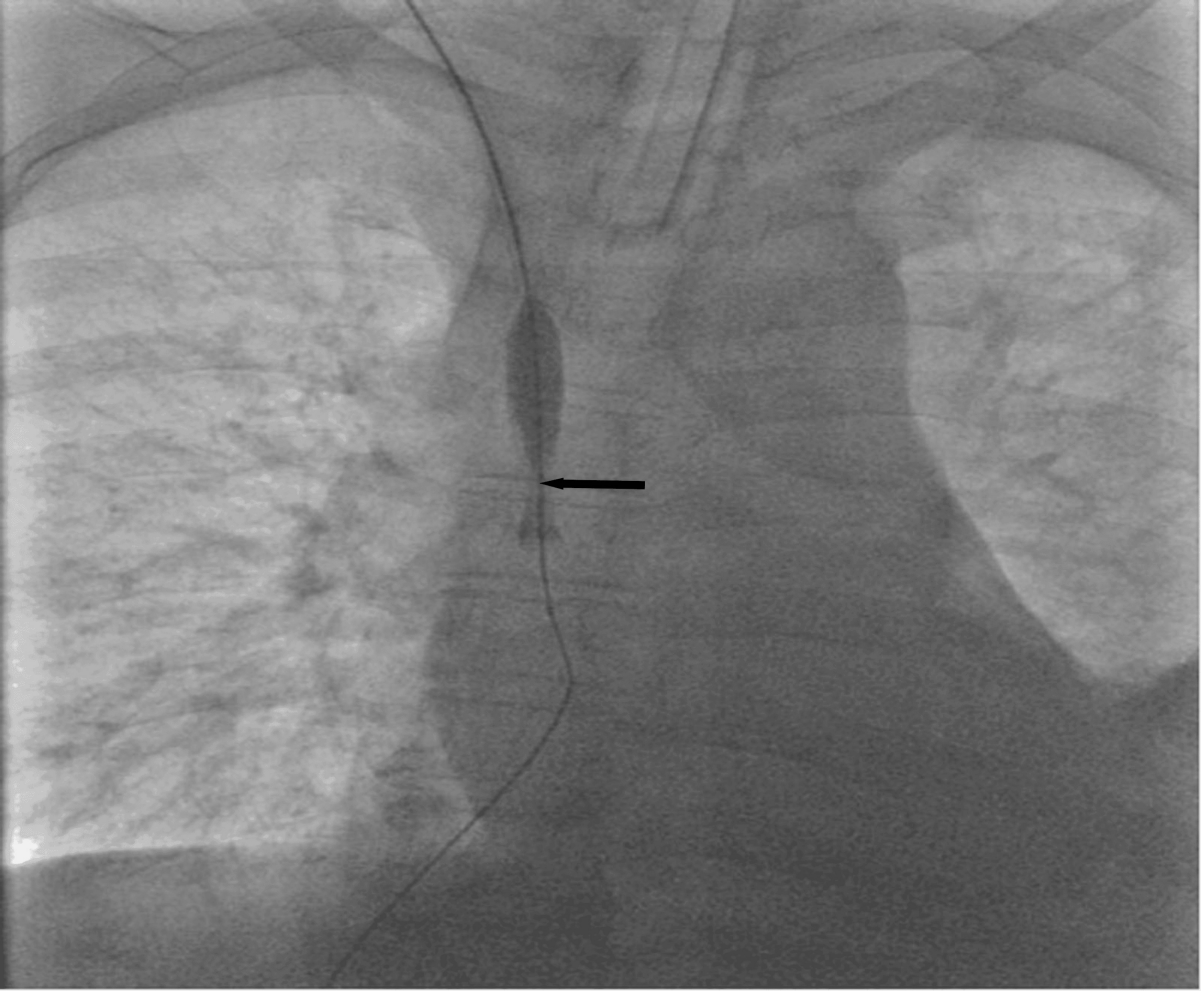
Balloon inflation. Serial balloon angioplasty of the occluded superior vena cava (anteroposterior view, right femoral venous approach). Inflation of an 8 mm × 60 mm balloon catheter reveals a characteristic waist deformity (arrow) at the site of maximal occlusion.

**Figure 4:**
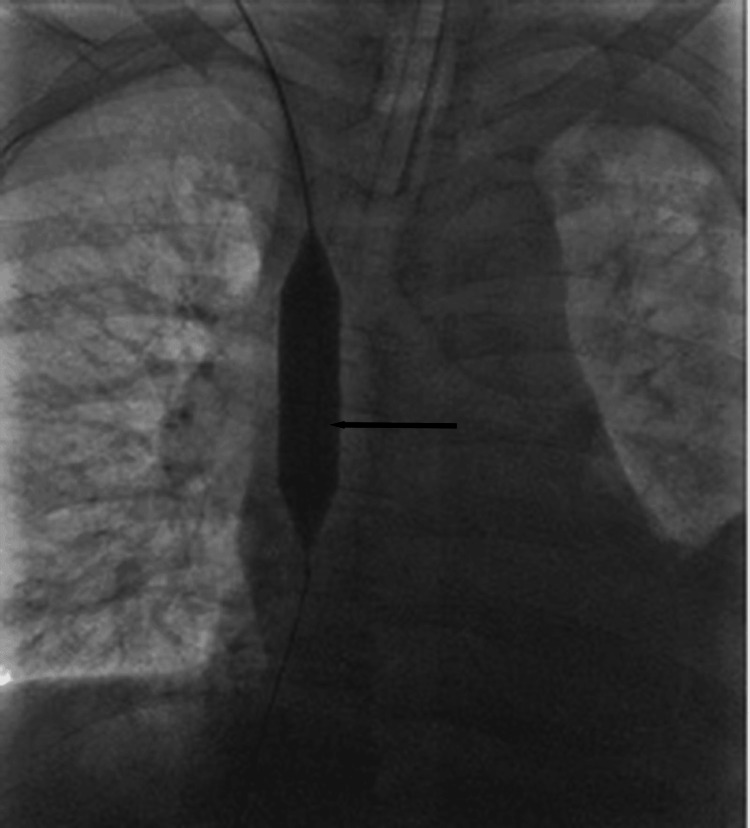
Post-angioplasty recanalization. Final balloon inflation within the occluded superior vena cava (anteroposterior view). An 18 mm × 60 mm balloon is fully inflated across the previously stenotic segment following serial dilatation. Complete effacement of the waist deformity is observed (arrow), indicating successful remodeling of the occluded segment before stent deployment.

**Figure 5:**
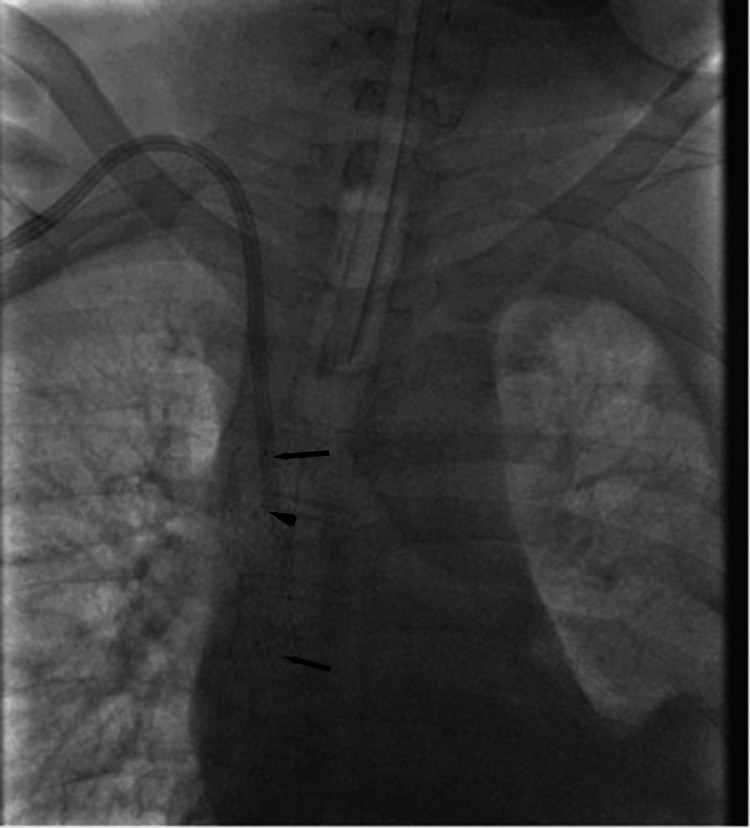
Final stent position. Post-procedural plain fluoroscopic image (anteroposterior view) following deployment of a 20 mm × 60 mm Venovo self-expanding stent (BD, Franklin Lakes, NJ). The stent is fully expanded and well-positioned across the previously occluded SVC segment, extending from the proximal SVC to the cavoatrial junction. Radiopaque markers at both ends (arrows) confirm accurate placement. The stent struts are well apposed to the vessel wall, with no evidence of migration, kinking, or fracture. The exchanged hemodialysis catheter tip resides within the stented segment (arrowhead). SVC, superior vena cava

Intraoperative fluoroscopy demonstrated complete SVC occlusion at the catheter tip (Figure 2), serial balloon inflation within the occluded segment revealing progressive effacement of the waist deformity (Figure 3), successful recanalization after angioplasty (Figure 4), and a well-positioned, widely patent stent post-deployment (Figure 5).

At three-month follow-up, the patient remained asymptomatic, with no further bleeding episodes, and maintained functional hemodialysis access through the revised catheter.

## Discussion

Our case of a 49-year-old man with ESRD on chronic hemodialysis who developed recurrent hematemesis from proximal esophageal varices (so-called *downhill *varices) highlights several important clinical lessons. First, it underscores the need for heightened suspicion of variceal bleeding secondary to central venous occlusion in patients without cirrhosis or portal hypertension. Second, it reinforces that endoscopic therapy alone is insufficient if the underlying venous occlusion is not corrected. Third, it identifies patients with long-term dialysis catheters as a high-risk group for central venous occlusion and its complications.

Although the incidence of downhill varices is rare in the general population, they may be underrecognized in high-risk settings. A systematic review of 41 cases by Ali et al. found that ESRD was the most common comorbidity (43.9%), with dialysis catheters or vascular grafts implicated in 51.2% of cases, indicating a shift from malignant to benign etiologies in modern series [1]. Downhill varices account for only 0.4%-10% of all esophageal varices and less than 1% of upper gastrointestinal bleeds [2]. From a nephrologist's perspective, central venous occlusion represents a major challenge in vascular access management, with prevention through fistula-first approaches being the optimal strategy [4].

The pathophysiology in dialysis patients involves chronic injury to the SVC by the catheter [3]. Recent studies highlight an increased incidence of such complications with longer catheter duration [3]. Loudin et al. reported that among hemodialysis-related bleeding from downhill varices, catheters are responsible for approximately 27% of cases [8]. Our case demonstrates this, as catheter dysfunction prompted imaging that revealed severe occlusion and collaterals. Labriola et al. found a 9.4% prevalence of SVC occlusion in patients with tunneled cuffed catheters, with diabetes and longer catheter duration as independent risk factors [6]. Echefu et al. further emphasized that central venous occlusion is a prevalent concern in dialysis vascular access management [9]. Our patient's risk factors - ESRD, catheter-dependent dialysis, diabetes, and hypertension - align closely with these findings.

Management focuses on hemostasis followed by etiology-specific therapy. Endoscopic band ligation provides effective acute control and is preferred over sclerotherapy to prevent complications such as embolism [3]. However, it offers only temporary relief unless SVC patency is restored. Guerrero-Macías et al. reported that percutaneous angioplasty with stenting achieves high success rates (95%-97%) in relieving occlusion and symptoms, leading to variceal regression [10]. Endovascular stenting for SVC syndrome has demonstrated excellent technical success and long-term patency in both malignant and benign cases [11]. Although much data derive from malignant SVC syndrome, benign catheter-related occlusion shows comparable efficacy with low recurrence and improved dialysis access [1]. In our patient, symptoms resolved after stenting, supporting this as definitive treatment.

Comparative cases illustrate similarities and contrasts. Ali et al. reported a 49-year-old hemodialysis patient with SVC occlusion and bleeding downhill varices managed successfully with band ligation alone, though stenting was not performed, carrying a theoretical risk of rebleeding [12]. Another report described a 34-year-old with catheter-induced SVC occlusion and recurrent hematemesis treated with vascular intervention [13]. A third case involved conservative management without banding or stenting due to the absence of active bleeding [14]. Horst et al. described SVC occlusion from a hemodialysis catheter leading to varices treated with stenting, with outcomes comparable to ours [15]. These cases highlight the heterogeneity in presentation and treatment, emphasizing the need for individualized management based on occlusion severity and bleeding risk.

Future efforts should focus on developing standardized guidelines for downhill varices, which are currently lacking, to optimize endoscopic and interventional timing [3]. In the dialysis population, preventive strategies, emphasizing arteriovenous fistulas, limiting catheter duration, and routine surveillance imaging, may reduce incidence. Multidisciplinary collaboration among gastroenterology, vascular surgery, and nephrology will improve outcomes, potentially aided by advanced imaging for early detection.

## Conclusions

This case demonstrates the diagnostic and therapeutic nuances of downhill esophageal varices in a hemodialysis patient with SVC occlusion. Prompt recognition through endoscopy and venography, along with band ligation for acute bleeding and stenting for definitive resolution, prevented recurrent hemorrhage. Comparative reports support the efficacy of vascular interventions, reflecting a shift toward benign etiologies. Future efforts should prioritize prevention and guideline development to reduce risk in vulnerable populations and improve outcomes in ESRD.
